# Effect of the Addition of Corn Husk Cellulose Nanocrystals in the Development of a Novel Edible Film

**DOI:** 10.3390/nano12193421

**Published:** 2022-09-29

**Authors:** David Choque-Quispe, Yudith Choque-Quispe, Carlos A. Ligarda-Samanez, Diego E. Peralta-Guevara, Aydeé M. Solano-Reynoso, Betsy S. Ramos-Pacheco, Fredy Taipe-Pardo, Edgar L. Martínez-Huamán, John Peter Aguirre Landa, Henrry W. Agreda Cerna, Julio C. Loayza-Céspedes, Miluska M. Zamalloa-Puma, Genaro Julio Álvarez-López, Alan Zamalloa-Puma, Elibet Moscoso-Moscoso, Yadyra Quispe-Quispe

**Affiliations:** 1 Water Analysis and Control Research Laboratory, Universidad Nacional José María Arguedas, Andahuaylas 03701, Peru; 2 Department of Agroindustrial Engineering, Universidad Nacional José María Arguedas, Andahuaylas 03701, Peru; 3 Research Group in the Development of Advanced Materials for Water and Food Treatment, Universidad Nacional José María Arguedas, Andahuaylas 03701, Peru; 4 Nutraceuticals and Biopolymers Research Group, Universidad Nacional José María Arguedas, Andahuaylas 03701, Peru; 5 Department of Environmental Engineering, Universidad Nacional José María Arguedas, Andahuaylas 03701, Peru; 6 Food Nanotechnology Research Laboratory, Universidad Nacional José María Arguedas, Andahuaylas 03701, Peru; 7 Department of Environmental Engineering, Universidad Tecnológica de los Andes, Andahuaylas 03701, Peru; 8 Department of Education and Humanities, Universidad Nacional José María Arguedas, Andahuaylas 03701, Peru; 9 Department of Business Administration, Universidad Nacional José María Arguedas, Andahuaylas 03701, Peru; 10 Departamento de Ingeniería Agropecuaria, Universidad Nacional de San Antonio Abad del Cusco, Andahuaylas 03701, Peru; 11 Department of Physics, Universidad Nacional de San Antonio Abad del Cusco, Cusco 08000, Peru; 12 Law and Humanities Faculty, Universidad Continental, Cusco 08000, Peru

**Keywords:** edible film, cellulose nanocrystals, potato starch, tensile strength, water activity, whiteness index

## Abstract

The cellulose from agroindustrial waste can be treated and converted into nanocrystals or nanofibers. It could be used to produce biodegradable and edible films, contributing to the circular economy and being environmentally friendly. This research aimed to develop an edible film elaborated with activated cellulose nanocrystals, native potato starch, and glycerin. The activated cellulose nanocrystals were obtained by basic/acid digestion and esterification with citric acid from corn husks. The starch was extracted from the native potato cultivated at 3500 m of altitude. Four film formulations were elaborated with potato starch (2.6 to 4.4%), cellulose nanocrystals (0.0 to 0.12%), and glycerin (3.0 to 4.2%), by thermoforming at 60 °C. It was observed that the cellulose nanocrystals reported an average size of 676.0 nm. The films mainly present hydroxyl, carbonyl, and carboxyl groups that stabilize the polymeric matrix. It was observed that the addition of cellulose nanocrystals in the films significantly increased (*p*-value < 0.05) water activity (0.409 to 0.447), whiteness index (96.92 to 97.27), and organic carbon content. In opposition to gelatinization temperature (156.7 to 150.1 °C), transparency (6.69 to 6.17), resistance to traction (22.29 to 14.33 N/mm), and solubility in acidic, basic, ethanol, and water media decreased. However, no significant differences were observed in the thermal decomposition of the films evaluated through TGA analysis. The addition of cellulose nanocrystals in the films gives it good mechanical and thermal resistance qualities, with low solubility, making it a potential food-coating material.

## 1. Introduction

The agroindustry generates organic wastes in large quantities that are not used or transformed [1]. Corn production generates corn leaves as harvest residue, and this material is mainly composed of cellulose, hemicellulose, and lignin. The extraction of cellulose from plant material may involve acid and basic or just one for hydrolysis treatments, whose product is widely used in manufacturing materials [2,3,4,5].

Nanocellulose hydrolyzed with strong acids (hydrochloric acid or sulfuric acid) has advantages over their untreated counterparts, especially in terms of thermal and mechanical stability [6,7,8], because they allow eliminating the amorphous state of cellulose, generating crystalline structures [9,10].

However, the use of acid treatments generates negative impacts on the environment. An alternative in producing cellulose monocrystals is to use less harmful organic acids (citric acid, oxalic acid, acetic acid, phosphoric acid, among others) [11,12], whose residues can be easily buffered. However, improving the removal of lignin and hemicellulose that contribute to the amorphous state could be limited. An alternative to overcome this disadvantage would be using methods combined with hydrolyses, such as microwave, ultrasound, high pressure, or plasma treatments, in addition to the use of lower concentration and quantity of acid during the hydrolysis of cellulose [13,14,15,16,17,18].

Cellulose nanocrystals are widely used in the food and pharmaceutical industry as a reinforcing agent, improving the viscosity and rheological behavior of emulsions, suspensions, and pastes [19,20]. In the same way, containers, films, and coatings manufactured for numerous products present an adequate mechanical, gas barrier, flexible, transparent, economical, and biodegradable properties [21,22].

These starches must have specific characteristics such as a good gelatinization point, mainly below 80 °C, to avoid retrogradation [23,24]. In addition, they must have good transmittance and transparency in solution or gel and high whiteness index [25,26]. Native potato starches grown above 3500 m of altitude seem to possess it [27].

However, the attributes of the film can be improved and modified by adding other constituents. They give it rigidity, torsional strength, surface uniformity, transparency, low water activity, resistance to solubility in different polar and non-polar solvent media, and good thermal behavior, depending on the purpose of its use [28,29,30,31,32].

Glycerin is one of the constituents that confers plasticity to the films. However [28,33], formulations have been tested, adding pectin, carrageenan, hydrocolloids, gums, and mucilage, among others. In addition, functional compounds such as fatty acids, essential oils, proteins, and bioactive compounds are added [23,28,34,35,36,37,38,39].

The use of new materials in the production of edible and biodegradable films encompasses the use of biological waste, including nanocelluloses [40,41,42,43], that act as fillers of the interstitial spaces in the polymeric matrix of the film, exchanging spaces occupied by other molecules [26,44], mainly water. In the same way, they grant particular behaviors, especially thermal and mechanical stability of the films, adjusting the solubility in aqueous media [40,45,46,47].

On the other hand, cellulose nanocrystals increase the availability of hydroxyl, carbonyl, and carboxyl functional groups, allowing them to establish hydrogen bonds with the polymeric matrix of the film [2,48], generating surfaces with more compact and uniform topography. Therefore, the research aimed to elaborate an edible film with native potato starch, cellulose nanocrystals, and glycerin, as well as the partial characterization of the physicochemical parameters, the study of thermal behavior, and infrared analysis.

## 2. Materials and Methods

### 2.1. Raw Material

The native potato (*Solanum tuberosum* subsp. Andigena) was collected from crop fields of the Centro Poblado Huancas, which is located in the district of Andarapa, Andahuaylas, Peru (13°40′42″ S, 73° 15′11″ W, and 3742 m of altitude), in the vegetative period from October 2020 to March 2021.

The corn wastes (husks) were collected from fields in the district of Andahuaylas, Peru (13°39′12″ S, 73°23′22″ W, and 2953 m of altitude), in the vegetative period from September 2020 to May 2021.

Chemical substances were used, such as glycerin (99% purity, food grade), anhydrous citric acid (99.5% purity), sodium hydroxide (98.5% purity), potassium permanganate (99.0% purity), acetic acid (99.8% purity), ethanol (96.9% *v/v*) from the Scharlau brand (Barcelona, Spain); also, sodium hypochlorite (14% solution), phenolphthalein and potassium bromide (IR Grade) of the Merck brand (Darmstadt, Germany).

### 2.2. Extraction of Starch and Cellulose Nanocrystals

The native potato starch was obtained by aqueous extraction and dried at 45 ± 2 °C for 14 h, then ground in a Resch model PM10 planetary mill (Haan, Germany) at 150 rpm for 3 min and sieved at 250 µm [27].

Corn husks were treated to remove lignin as follows: corn husks were minced to 1 mm, 2.5 g were weighed to transfer to 3 quartz digestion tubes, and 45 mL of NaOH at 2% was added. Then, it was left to soak for 10 min and was completed to 50 mL with NaOH at 2%. After that, it was hydrolyzed by digestion for 20 min in an SCP Science brand microwave digester (Quebec, QC, Canada) two times. The samples were then rinsed with distilled water to neutral pH.

Subsequently, the hemicellulose was removed through a bleaching process with 50 mL of sodium hypochlorite at 0.525% and stirring at 100 rpm for 30 min. Afterward, the samples were rinsed until pH 7, and the removal was contrasted with phenolphthalein.

The bleached samples were hydrolyzed with 30 mL of citric acid at 3% through a microwave digester for 20 min at 140 °C with a ramp of 15 min of heating and 10 min of cooling. Immediately, samples were rinsed until pH 7 and checked with potassium permanganate solution until coloration was no longer present.

The hydrolyzed samples were dried at 60 °C for 18 h and ground to 250 µm in a cyclone mill, Twister model, Retchsh brand (Haan, Germany). Immediately, it was taken to another grinding sieve on a mesh No. 325 (<45 µm) in a planetary ball mill, model PM100, Retsch brand (Haan, Germany), obtaining activated cellulose nanocrystals (NCCA).

### 2.3. Elaboration of the Films

**Figure 1:**
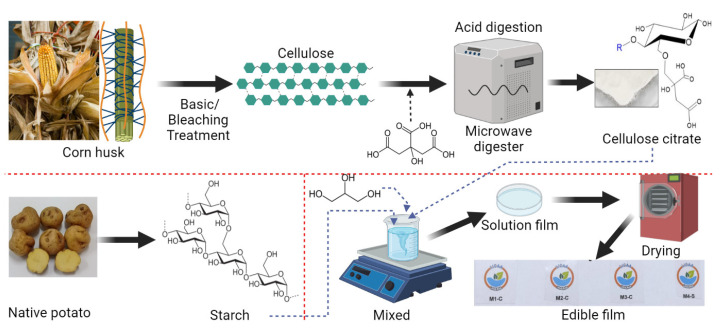
Films elaboration diagram.

Films were elaborated by thermoforming, following the methodology proposed by Choque-Quispe et al. [27], according to the formulation shown in Table 1 and Figure 1.

### 2.4. NCCA SEM Analysis

The morphology of the cellulose and NCCA was visualized through a scanning electron microscope (SEM), model Prism E, Thermo Fisher (Massachusetts, MA, USA), at an acceleration voltage of 25 kV and a magnification of 1000X.

### 2.5. NCCA Particle Size

An amount of 4 mg of NCCA was dispersed in 5 mL of ultrapure water, stirred at 1000 rpm for 5 min, then ultrasonicated for 10 min. An aliquot was taken in a capillary, and the hydrodynamic distribution of diameters and sizes was determined by dynamic light scattering (DLS) using the Nicomp brand equipment, nano ZLS Z3000 (Massachusetts, MA, USA).

### 2.6. Water Activity (a_w_)

Raw material samples (S and NCCA) and films (1 cm × 1 cm) were taken and placed in a conditioning chamber at 43 ± 2% RH and room temperature (20 °C on average) for 24 h. Afterward, previously calibrated the *a_w_* was determined in Rotronic brand equipment, model HygroPalm23-AW (Bassersdorf, Switzerland).

### 2.7. Color

The color characteristics of films were measured using the Konica Minolta colorimeter, model CR-5 (Tokyo, Japan), luminosity L* was determined (0 = black and 100 = white), chroma a* (+*a* = red, −*a* = green), chroma b* (+*b* = yellow and −*b* = blue) [49,50].

In the same way, the color difference (ΔE*) (Equation (1)), yellowness (YI) (Equation (2)), and whiteness indexes (WI) (Equation (3)) [38,51].

(1) $$\Delta E^{*}=\sqrt{{\left(L^{*}-L\right)}^{2}+{\left(a^{*}-a\right)}^{2}+{\left(b^{*}-b\right)}^{2}},$$

(2) $$YI=\frac{142.86xb^{*}}{L},$$

(3) $$WI=100-\sqrt{{\left(100-L^{*}\right)}^{2}+{\left(a^{*}\right)}^{2}+{\left(b^{*}\right)}^{2}},$$

where *L, a, b* are the color parameter values of white standard (*L* = 91.83, *a* = −0.73, *b* = 1.52), and L*, a*, b* are the color parameter values of the sample.

### 2.8. Transparency

The emulsions were molded on glass plates at room temperature for 24 h, obtaining coating films. The films were conditioned in a quartz vial with a rectangular side, and the transmittance was read at 600 nm in a Thermo Fisher UV–Vis spectrophotometer, model Genesys 150 (Madison, WI, USA) [52]. Transparency was reported as the ratio between transmittance and thickness (nm/mm).

### 2.9. Solubility

Films were cut in 2 × 2 cm, preconditioned at 0% RH for seven days, and weighed (initial weight). Then, it was placed in a Beaker with 50 mL of distilled water, commercial ethanol at 96% *v/v*, sodium hydroxide at 1.0 M, and 1.8 M of acetic acid at 25 °C and stirred at 40 rpm for 24 h. Subsequently, it was filtered through Whatman No. 41 paper, and the filtrate was dried at 60 °C until constant weight (final weight). The total soluble material was calculated according to Equation (4) [52,53].

(4) $$\%\;Solubility=\frac{initial\;weight-final\;weight}{initial\;weight}\times 100,$$

### 2.10. Total Carbon Determination

Total organic carbon (TOC) was determined, weighing 50 mg of raw materials and films, and they were placed in ceramic crucibles and covered with fiberglass. Then, they were taken to the TOC module, Shimadzu brand model TOC–L CSN–SSM 5000A (Kyoto, Japan), with an oxygen flow of 150 mL/min. The samples were subjected to 900 °C in a combustion chamber, and the results were reported in triplicate through the TOC control L V. 1.07 software (Kyoto, Japan).

### 2.11. IR Analysis

Pressed tablets were prepared with 0.1% of the film in KBr (IR Grade, Darmstadt, Germany). They were taken to the transmission module of the FTIR spectrometer (Fourier transform infrared spectroscopy), Thermo Fisher (Waltham, MA, USA), Nicolet IS50 model, in a range of 4000 to 400 cm^−1^ with a resolution of 4 cm^−1^.

### 2.12. Tensile Strength Analysis

The films were cut into 7 × 2 cm in rectangular strips and taken to materials testing machine, Pasco model ME-8236 (California, CA, USA), in order to determine the tensile strength. The tests were carried out five times, following the methodology proposed by ASTM D882-12. The results were reported as the ratio of the maximum breaking strength (N) and thickness (mm).

### 2.13. Film Thickness

The thickness was measured using a digital micrometer, model IP65, brand Mitutoyo (Kawasaki, Japan), with a sensitivity of 0.001 mm. Strips of 1 cm × 3 cm films were cut and placed between two glass plates. The thickness was determined by the difference between the thickness of the glass plates without a sample and the plates with a sample. Measurements were made for three different strips.

### 2.14. Thermal Analysis

The thermal stability of raw materials and films was determined by thermogravimetric analysis (TGA). The samples were loaded in alumina crucibles (Al_2_O_3_) and taken to a TA Instruments brand equipment, model TGA550, with Trios V software (Delaware, DE USA), with a range from 20 to 600 °C, with a heating rate of 10 °C/min and nitrogen supply of 50 mL/min.

The thermal transition properties from raw materials and films were analyzed through a differential scanning calorimeter (DSC), TA Instruments brand, model DSC2500 (Waters TM, New Castle, DE, USA), under a nitrogen atmosphere (50 mL/min). Samples were sealed in an aluminum pan and scanned from 20 to 200 °C at a heating rate of 5 °C/min. The equipment was stabilized through a baseline run at analysis conditions for 1 h.

### 2.15. Statistical Analysis

The results were analyzed through a one-way ANOVA and Tukey’s multiple comparison test at 5% significance.

## 3. Results and Discussions

### 3.1. Synthesis of Activated Cellulose Nanocrystals (NCCA)

Cellulose was obtained from corn wastes (husks), after lignin and hemicellulose were removed with sodium hydroxide and sodium hypochlorite, respectively.

**Figure 2:**
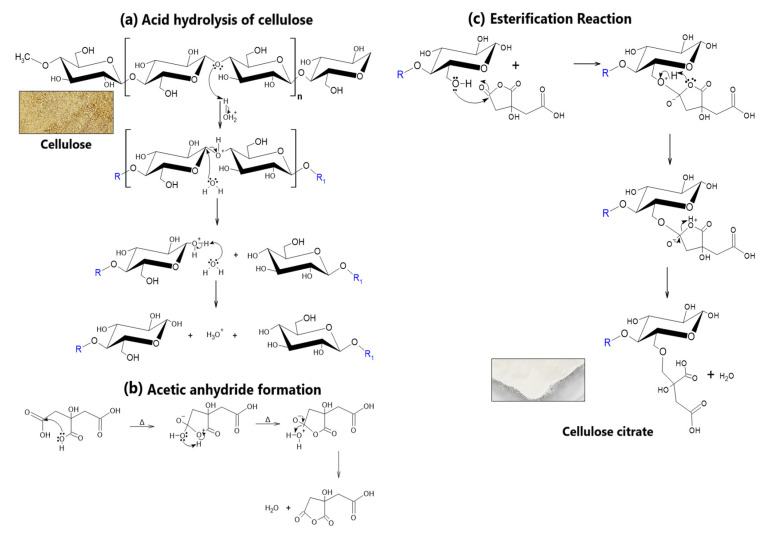
(**a**) Corn husk cellulose and acid hydrolysis reaction, (**b**) formation of citric anhydride, (**c**) esterification reaction and cellulose citrate (NCCA).

NCCA was obtained through two chemical processes from cellulose. First, acid hydrolysis of the amorphous region of cellulose was performed [54,55,56,57], which was treated with citric acid by microwave digestion, allowing the breaking of the β-1,4 glycosidic bond (Figure 2a). An esterification reaction occurs immediately between the citric anhydride (Figure 2b) linking with hydrolyzed cellulose units, obtaining cellulose citrate as a product (Figure 2c), and getting NCCA. Acid treatments at high temperatures allow the formation of nanocrystals [58,59,60,61,62], although, with treatments in a strong acid medium such as HCl and H_2_SO_4_, digestion takes up to 13 h [2,62,63,64]. In the study, the production of NCCA was carried out by microwave digestion at 140 °C for 20 min, obtaining particles of 676.0 nm, on average, in an aqueous solution.

### 3.2. SEM Morphology of the NCCA

**Figure 3:**
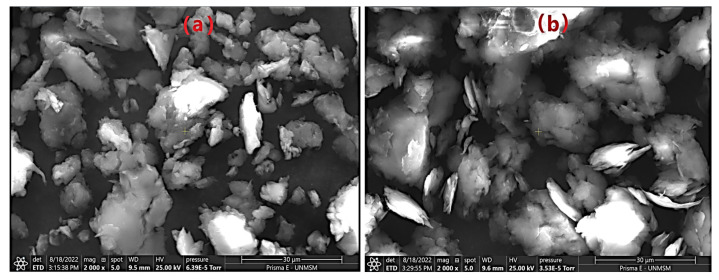
SEM images (**a**) cellulose, (**b**) NCCA.

Figure 3 shows photomicrographs of the morphology of untreated and treated cellulose (NCCA). Cellulose has a compact irregular shape (Figure 3a), mainly due to the presence of lignin, hemicellulose, and pectins [22,65,66]. This structure was significantly modified due to the alkali treatment and bleaching with sodium hypochlorite, removing non-cellulosic constituents [16,20,67]; the addition of citric acid allowed the formation of nanocrystals with a sharp lamellar shape at the contours (Figure 3b).

On the other hand, the treatment of the combined effect of temperature (140 °C)-microwaves helps in the rupture of cellulosic fibers and the detachment of non-cellulosic constituents, allowing the formation of nanocrystals. These results have been obtained in numerous trials when treated with strong acids and long digestion times [13,14,17,18,19,47,68,69]; however, the present investigation shows the feasibility of obtaining NCCA in less time and with an organic acid, demonstrating an alternative to obtaining it.

### 3.3. Water activity (a_w_)

*a_w_* allows knowing the hygroscopic capacity of materials, making it an essential parameter in food preservation [70], due to the active receptor sites of water molecules on its surface [71]. It was observed that S reported lower values (0.252 ± 0.003) (Table 2), although cellulose and NCCA show similar values. This would be due to the low availability of active sites capable of binding water molecules [71,72].

**Figure 4:**
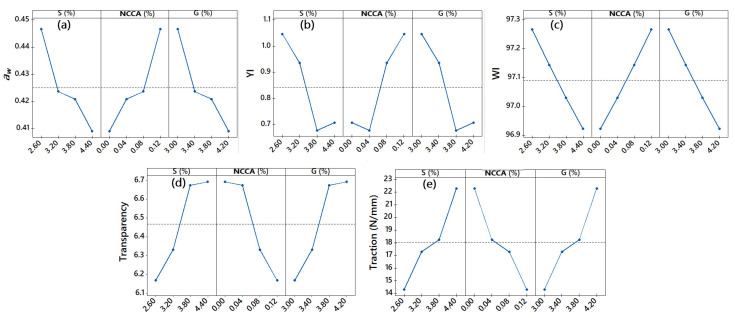
Main effects for (**a**) *a_w_*, (**b**) yellowness index, (**c**) whiteness index, (**d**) transparency, (**e**) traction.

Regarding films, it was observed that the addition of G and S considerably decreases the *a_w_* (Figure 4a), while the increase in NCCA in the formulations considerably increases the *a_w_*. This could be due to the fact that the polymeric chains of NCCA present a greater availability of hydroxyl groups compared to S and G.

### 3.4. Color

Color is an essential aspect of the visual quality of biodegradable films, and they are desirable when they allow the natural color of coated food to be transmitted [48].

It was observed that luminosity L* of NCCA increases considerably compared to cellulose, although with a higher value for S (Table 3), while relatively high values were observed for films greater than 96.96, although they show a significant difference (*p*-value < 0.05). On the other hand, a* chroma values are close to zero, and the tendency cannot be defined, while b* tends to low yellow.

Regarding YI, cellulose reported a higher value, although it decreased considerably for NCCA (Table 3). This is due to the removal of lignin and hemicellulose during acid and alkaline activation. For films, YI increases with the addition of NCCA (Figure 4b), while the addition of G and S decreases considerably. So, YI would be conditioned to the addition of NCCA and G, films with slightly yellow tendencies being desirable for use as a food coating [35,38,48,73]. On the other hand, the WI for the films (*p*-value < 0.05) increases considerably with the addition of NCCA, the opposite happens with the addition of S and G (Figure 4c). ΔE reports values between 5.29 and 5.65 (*p*-value < 0.05), and it increases with the addition of NCCA and G (Table 3). This is a characteristic behavior of the addition of plasticizers [38,74,75], although it could be affected by the color of the raw materials of films [38,72].

### 3.5. Transparency

The transparency of edible films used as coatings allows the integrity of the visual quality on the surface of a food to be transmitted. When this is greater, it will allow for the presentation of the product naturally and minimize oxidative degradation as well [74,76].

**Figure 5:**
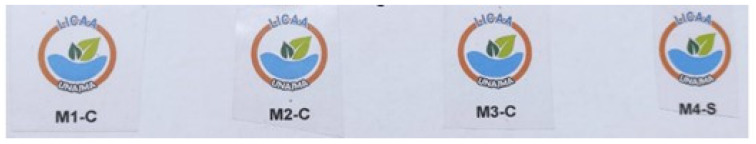
Transparency of films on images.

It was observed that transmittance reported values between 77.75 and 84.33%, while the transparency was between 6.17 and 6.69 nm/mm (*p*-value < 0.05) (Table 4). Films elaborated with different formulations were highly transparent (Figure 5), making them a potential material to use as a food coating.

The addition of NCCA considerably decreased transparency (Figure 4d). In contrast, the addition of S and G reported an opposite behavior. It would be because G forms insoluble nanoaggregates G-NCCA and G-S during the thermoforming and drying of the films, which formed immiscible phases, with new interactions in the reorganization of the three-dimensional network of the gelled starch matrix [48,77,78,79].

### 3.6. Solubility

Solubility is essential in edible biodegradable films for food and pharmaceutical packaging [38,39,80]. It was observed that films in an acid medium reported lower solubility (between 8.87 and 9.53%) compared to those subjected to ethanol, sodium hydroxide, and water (Table 5); likewise, films that did not contain NCCA reported higher solubility in water, ethanol and sodium hydroxide. However, the addition of NCCA and G considerably influences the solubility of the films (*p*-value < 0.05).

The native potato starch-G interaction allows obtaining polymeric matrices that are more resistant to polar solvent media due to the plasticity effect that these raw materials present [27,81], although the amylose contribution from starch has a considerable influence [82,83,84]. The addition of NCCA allows three-dimensional matrices to be configured in the films, providing the most significant number of hydroxyl and carbonyl groups. In addition, allowing water molecules to be trapped on the surface generates a barrier effect, resulting in lower solubility in polar media [82,85], although solubilities up to 90% have been reported in films elaborated with chia seeds and glycerol [75,86]. Therefore, films elaborated with S, NCCA, and G would present better resistance to solubility in weak acid media.

### 3.7. Organic Matter of Raw Materials and Films

**Figure 6:**
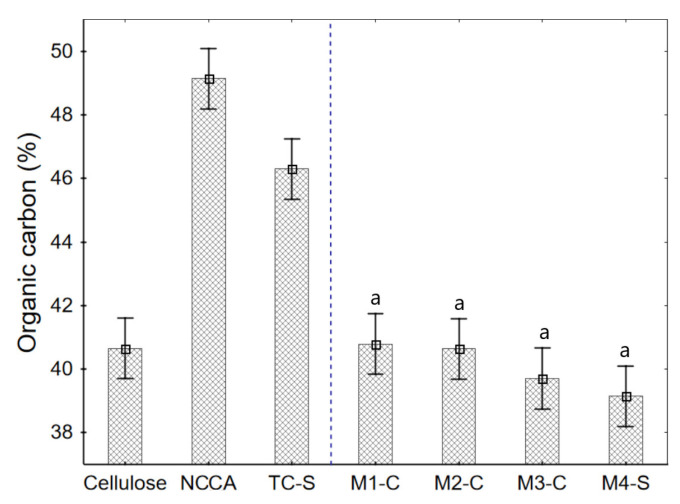
Organic carbon content in raw materials and films.

The acid and basic treatment of the cellulose allows for the removal of some constituents such as fats and proteins [57,58,63], and at the same time, when activated with citric acid increases the number of carbons; for this reason, a considerable increase in organic carbon in NCCA was observed (Figure 6). Thus, the films slightly increase the organic carbon content (although this is insignificant, *p*-value > 0.05). It is mainly due to the contribution of NCCA (49.14%) and S (47.27%). Therefore, films produced have a good content of organic carbon (around 40%), making them biodegradable.

### 3.8. IR Structural Analysis of Raw Materials and Films

The FTIR analysis makes it possible to show the arrangement of the functional groups of raw materials (S, NCCA, and G) and how these are modified during the thermal process of elaboration of the biodegradable films, which shows the interactions of the stretching, bending, and torsion type of chemical bonds in the film matrix network [27,87].

**Figure 7:**
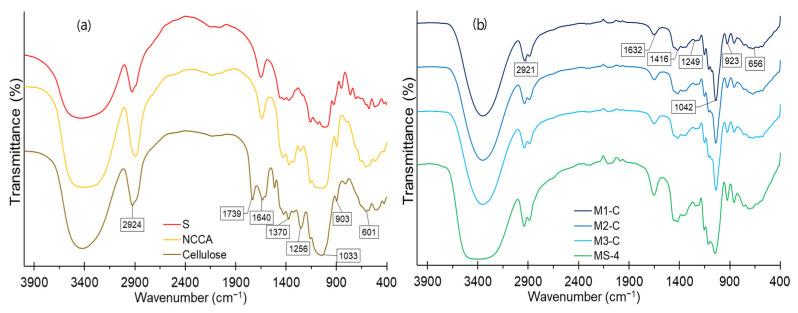
(**a**) FTIR spectrum for raw materials, (**b**) FTIR spectrum for films.

The FTIR analysis showed a broad band around 3400 cm^−1^ corresponding to an -OH stretching of water and carbohydrate molecules with greater intensity for S (Figure 7a). This fact affects the films, especially in MS-4 (higher content of S and G), where the spectrum is broader compared to other formulations (Figure 7b). The peak around 2900 cm^−1^ is attributed to the C-H vibrations of methyl groups of esters, with greater intensity for NCCA (derived from the esterification reaction producing cellulose citrate). As for S and cellulose, it is attributed to the vibrations of the C-H extension of the methyl groups of their structures. While in the films, it is observed that MS-4 showed greater intensity for this spectrum. It would be due to the esterification of glycerin [48]. The peak at 1739 cm^−1^ in the cellulose corresponds to C=O ester bonds which disappear for NCCA, due to the acid and basic treatment during its activation, but the films show them. Around 1640 cm^−1^, a vibration of C=O stretching and -OH stretching of the water present occurs with greater intensity for S and MS-4 film, giving it greater hygroscopicity [29,88,89].

On the other hand, around 1400 cm^−1^, the C-O, C-H, and -OH stretching is manifested, mainly corresponding to the polymeric chains of carbohydrates with greater intensity for S and MS-4. The high-intensity peaks close to 1040 cm^−1^ correspond to the C-O, C-O-C, and C-OH stretching of esters, carbohydrates, and glycerin with greater intensity for NCCA and MS-4. These groups are responsible for the dipole–dipole interactions between the constituents of the film [27,37]. While the area below 800 cm^−1^ “fingerprints” is attributed to the C-H and C-O stretching of starches, cellulose, and sugars [90,91,92].

### 3.9. Film Tensile Strength Analysis

The tensile strength of edible films depends on the formulation, constitution, and production method. It is an essential parameter, primarily if the films are used as a food coating material [35,48].

The films showed thicknesses between 0.12 to 0.15 mm (Table 6), observing an increasing trend with the decrease in NCCA, although not significant (*p*-value > 0.05). This difference is due to the amount of water added to the formulation, which is eliminated during drying. In the same way, it is due to the agglomeration capacity of the starch [48,93].

The tensile strength of films ranges from 14.33 to 18.24 N/mm (Table 6), observing greater resistance with the addition of G and S (Figure 4d), while NCCA makes it significantly decrease (Figure 4d). It would be due to the fact of gelatinization capacity that starch presents, and plasticizing capacity of glycerin [39,94], while NCCA acts as an interstitial filler of the polymeric matrix, which would generate weak electrostatic and chemical attraction with other constituents, resulting in less traction [75,95].

### 3.10. Thermal Analysis of Raw Materials and Films

**Figure 8:**
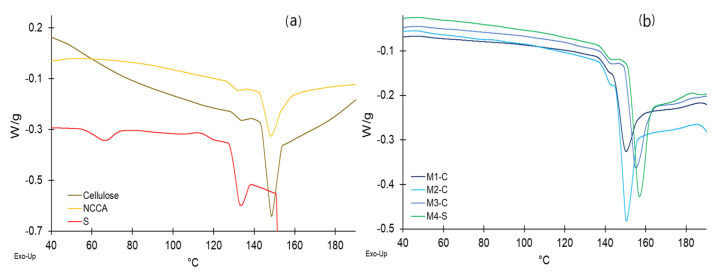
DSC thermogram, (**a**) raw materials, (**b**) films.

The thermal behavior evaluated through DSC analysis of films shows a slight increase in the glass transition temperature (Tg) with the addition of S and G, from 141.3 to 142.3 °C, although with greater intensity for the enthalpy (Table 7), showing endothermic peaks (Figure 8b). On the other hand, the melting temperature (Tm) reported values between 150.1 and 156.7 °C, and their respective melting enthalpies were between 11.49 and 19.06 J/g, with high-intensity endothermic peaks. This peak would be influenced mainly by the intensity of S (Figure 8a), whose energy contribution was 2804.90 J/g.

It would be mainly due to the energy contribution of S (9.66 J/g) and G [25] since the starch when exceeding 60 °C, presents an irreversible swelling, achieving the destructuring of its granules, and a highly stable glassy structure, with strong molecular movement in transition to melting [80,96,97]. It establishes strong bonds due to the hydroxyl groups [27,37,39], which is why this material is widely used in the production of edible films [24,29,80]. The fact that the addition of NCCA decreases Tg and Tm and it would be because these nanocrystals when lodged in the interstitial spaces, agglomerate as fillers and act as thermal conductors, which is evidenced by low values of ΔH for NCCA and films [37,98].

**Figure 9:**
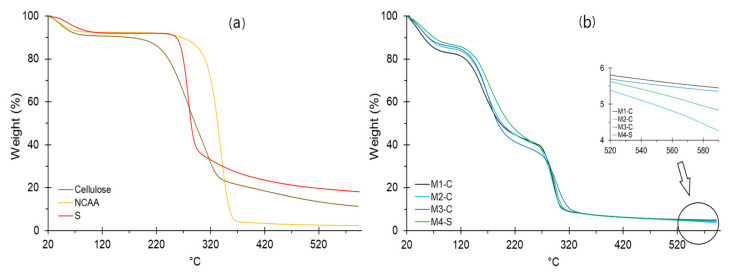
TGA thermogram for (**a**) raw materials, (**b**) films.

Regarding the thermal stability of films, it was observed that they present four well-defined thermal stages (Figure 9b). The first stage occurs up to 100 °C. The weight loss decreases with the increase in S and G from 17.17 to 13.08% (Table 8). The loss is due to the chemisorbed water of the polymeric matrix, whose molecules are linked to carbonyl, carboxyl, and hydroxyl functional groups (mainly from starch), through hydrogen bonds as free water and weakly bound water [23]. This behavior is related to *a_w_* (Table 2). A second stage occurs until 220 °C. In this stage, the mass loss is mainly due to the volatilization and combustion of glycerin (plasticizer) [23,97,99,100], which would be confirmed by the increase in the percentage of loss for films M1-C to M3-C (Table 8), who have higher glycerin content, respectively.

A third stage occurs until 320 °C on average. It is related to NCCA and S polysaccharides’ decomposition, representing between 30.93 and 38.03% (Table 8). It is because films are influenced by NCCA content, showing an increase from M3-C to M1-C. It can be confirmed by the higher mass loss (85.83%) that NCCA presents in the second stage (Figure 9a). The fourth stage is linked to the total decomposition of NCCA and S residues, whose content refers to the carbon generated above 320 °C. Although there is no evidence of a sequential behavior with S or NCCA, it can be concluded that the S content attributes a higher carbon content, which is appreciated from 520 °C (Figure 9b).

## 4. Conclusions

Cellulose nanocrystals (NCCA) obtained from corn husk by hydrolysis with citric acid and assisted by microwaves were used in combination with potato starch and glycerin to elaborate edible films by thermoforming. The NCCA allowed increasing the presence of hydroxyl, carbonyl, and carboxyl groups. In the same way, it significantly influenced the *a_w_*, TOC, YI, and WI of the films, increasing their values, while transparency, solubility in aqueous media, and tensile strength decreased significantly (*p*-value < 0.005). The melting temperature, Tm, decreases considerably with the addition of NCCA, allowing greater water retention. The properties of the elaborated films suggest a material with potential qualities for its application as an alimentary coating, being environmentally friendly, and contributing to the circular economy.

## Figures and Tables

**Table 1:** Formulation of biodegradable films.

Formulation	Starch (S) (%)	NCCA (%)	Glycerin (G) (%)	Water (%)
M1-C	2.60	0.12	3.00	94.28
M2-C	3.20	0.08	3.40	93.32
M3-C	3.80	0.04	3.80	92.36
M4-S	4.40	0.00	4.20	91.40

**Table 2:** *a_w_* of raw material and films.

Material	$\bar{x}$	±	SD	CV (%)	*	T (°C)
Cellulose	0.404	±	0.001	0.21		20.7
NCCA	0.398	±	0.004	1.06		23.4
S	0.252	±	0.003	1.23		20.0
M1-C	0.447	±	0.003	0.66	a	20.4
M2-C	0.424	±	0.004	0.83	b	20.8
M3-C	0.421	±	0.005	1.26	b	20.9
M4-S	0.409	±	0.007	1.62	c	21.0

Where $\bar{x}$, is the arithmetic mean; SD is the standard deviation; CV; variability coefficient. * Evaluated through an ANOVA and Tukey’s test at 5% significance, for *n* = 5.

**Table 3:** Color L*, a*, b*, YI, and WI of raw material and films.

Material	L*					a*					b*				
	$\bar{x}$	±	SD	CV (%)	*	$\bar{x}$	±	SD	CV (%)	*	$\bar{x}$	±	SD	CV (%)	*
Cellulose	69.46	±	0.19	0.27		0.04	±	0.01	13.32		10.56	±	0.09	0.88	
NCCA	84.10	±	0.07	0.08		0.94	±	0.01	0.61		4.84	±	0.02	0.32	
S	92.10	±	0.01	0.01		−0.02	±	0.01	34.64		2.79	±	0.01	0.21	
M1-C	97.36	±	0.02	0.02	a	0.11	±	0.01	9.09	a	0.71	±	0.02	2.14	a
M2-C	97.22	±	0.02	0.02	b	0.08	±	0.01	6.93	b	0.64	±	0.01	1.81	b
M3-C	97.07	±	0.02	0.02	c	0.04	±	0.01	13.32	c	0.46	±	0.01	2.17	c
M4-S	96.96	±	0.01	0.01	d	0.03	±	0.01	17.32	c	0.48	±	0.01	2.08	c
	**YI**					**WI**					ΔE*				
	** $\bar{x}$ **	**±**	**SD**	**CV (%)**	*	** $\bar{x}$ **	**±**	**SD**	**CV (%)**	*	** $\bar{x}$ **	**±**	**SD**	**CV (%)**	*
Cellulose	21.73	±	0.24	1.10		67.69	±	0.20	0.30		24.14	±	0.20	0.84	
NCCA	8.22	±	0.03	0.37		83.36	±	0.07	0.08		8.57	±	0.06	0.73	
S	4.32	±	0.01	0.21		91.63	±	0.01	0.01		1.48	±	0.00	0.14	
M1-C	1.05	±	0.02	2.13	a	97.27	±	0.01	0.01	a	5.65	±	0.01	0.21	a
M2-C	0.94	±	0.02	1.82	b	97.14	±	0.02	0.02	b	5.52	±	0.02	0.39	b
M3-C	0.69	±	0.01	2.17	c	97.03	±	0.02	0.02	c	5.40	±	0.02	0.38	c
M4-S	0.71	±	0.01	2.09	c	96.92	±	0.01	0.01	d	5.29	±	0.01	0.22	d

Where $\bar{x}$, is the arithmetic mean; SD is the standard deviation; CV; variability coefficient. * Evaluated through an ANOVA, different letters indicate significant differences, determined through Tukey’s test at 5% significance, for *n* = 5.

**Table 4:** Film transparency.

Film	Transmittance (%)					Transparency (nm/mm)				
	$\bar{x}$	±	SD	CV (%)	*	$\bar{x}$	±	SD	CV (%)	*
M1-C	77.75	±	0.55	0.70	a	6.17	±	0.04	0.70	a
M2-C	79.80	±	0.66	0.83	a	6.33	±	0.05	0.83	a
M3-C	83.43	±	0.59	0.70	b	6.67	±	0.05	0.70	b
M4-S	84.33	±	0.39	0.46	c	6.69	±	0.03	0.46	c

Where $\bar{x}$, is the arithmetic mean; SD is the standard deviation; CV; variability coefficient. * Evaluated through an ANOVA, different letters indicate significant differences, determined through Tukey’s test at 5% significance, for *n* = 5.

**Table 5:** Film solubility.

Film	Ultrapure Water					Ethanol (96% *v*/*v*)					Sodium Hydroxide (0.10 M)					Acetic Acid (0.18 M)				
	$\bar{x}$	±	SD	CV (%)	*	$\bar{x}$	±	SD	CV (%)	*	$\bar{x}$	±	SD	CV (%)	*	$\bar{x}$	±	SD	CV (%)	*
M1-C	38.06	±	1.00	2.63	a	39.48	±	0.65	1.64	a	42.34	±	0.40	0.94	a	9.53	±	0.24	2.55	a
M2-C	38.78	±	1.12	2.89	a,b	39.53	±	0.65	1.65	a	38.27	±	0.25	0.67	b	8.87	±	0.39	4.38	b
M3-C	40.64	±	0.65	1.59	b	42.25	±	0.47	1.10	b	38.51	±	0.46	1.19	b	8.91	±	0.05	0.58	a,b
M4-S	40.11	±	0.41	1.01	a,b	42.36	±	0.38	0.89	b	37.70	±	0.46	1.22	b	9.02	±	0.20	2.17	a,b

Where $\bar{x}$, is the arithmetic mean; SD is the standard deviation; CV; variability coefficient. * Evaluated through an ANOVA, different letters indicate significant differences, determined through Tukey’s test at 5% significance, for *n* = 5.

**Table 6:** Film thickness and traction.

Film	Thickness (mm)					Traction (N/mm)				
	$\bar{x}$	±	SD	CV (%)	*	$\bar{x}$	±	SD	CV (%)	*
M1-C	0.12	±	0.01	4.68	a	14.33	±	0.50	3.49	a
M2-C	0.12	±	0.01	4.68	a	17.30	±	0.17	0.98	b
M3-C	0.15	±	0.01	6.67	a	18.24	±	0.89	4.88	b
M4-S	0.14	±	0.01	4.22	a	22.29	±	0.96	4.32	c

Where $\bar{x}$, is the arithmetic mean; SD is the standard deviation; CV; variability coefficient. * Evaluated through an ANOVA, different letters indicate significant differences, determined through Tukey’s test at 5% significance, for *n* = 3.

**Table 7:** DSC thermal transitions for raw material and films.

Material	Tp (°C)	ΔH (J/g)	Tg (°C)	ΔH (J/g)	Tm (°C)	ΔH (J/g)
Cellulose	---	---	131.4	1.11	148.1	16.03
NCCA	---	---	133.4	1.21	148.5	21.40
S	66.1	5.31	132.9	9.66	153.4	2804.90
M1-C	---	---	141.3	0.07	150.1	11.49
M2-C	---	---	142.1	0.45	150.4	16.07
M3-C	---	---	142.3	0.71	154.9	17.41
M4-S	---	---	142.3	0.84	156.7	19.06

**Table 8:** Weight loss and decomposition temperature.

Material	First Stage		Second Stage		Third Stage		Fourth Stage		Residue (%)
	Weight Loss (%)	T (°C)	Weight Loss (%)	T (°C)	Weight Loss (%)	T (°C)	Weight Loss (%)	T (°C)	
Cellulose	11.23	80.0	65.77	352	11.55	590			11.45
NCCA	7.784	80.0	85.83	362	4.07	590			2.316
Starch	5.72	110	55.95	312	17.12	590			21.21
M1-C	17.17	99	38.06	220	35.78	320	4.04	590	4.94
M2-C	14.87	100	40.23	220	35.68	319	5.41	590	3.81
M3-C	14.01	98	44.50	220	30.93	318	5.71	590	4.85
M4-S	13.08	99	39.67	220	38.03	320	4.91	590	4.31

## Data Availability

The data presented in this study are available in this same article.
